# Deciphering Depressive Mood in Relapsing-Remitting and Progressive Multiple Sclerosis and Its Consequence on Quality of Life

**DOI:** 10.1371/journal.pone.0142152

**Published:** 2015-11-10

**Authors:** Delphine Lamargue Hamel, Mathilde Deloire, Aurélie Ruet, Julie Charré-Morin, Aurore Saubusse, Jean-Christophe Ouallet, Bruno Brochet

**Affiliations:** 1 INSERM U.862, Neurocentre Magendie, Université de Bordeaux, Bordeaux, France; 2 INSERM-CHU CIC-P 0005, & Service de Neurologie, CHU de Bordeaux, F-33076, Bordeaux, France; Charité University Medicine Berlin, GERMANY

## Abstract

**Background:**

Depressive mood and other emotional symptoms are common in multiple sclerosis (MS). The patient-reported outcome version of the “Echelle d’Humeur Dépressive” (EHD-PRO) aims to differentiate between two dimensions of depressive mood in people living with MS (PwMS).

**Objectives:**

First, to compare EHD-PRO assessment and its two dimensions, lack of emotional control and emotional blunting, between a large sample of healthy controls (HCs) and two samples of PwMS, relapsing-remitting MS (RRMS) and primary progressive MS (PPMS); and second, to analyse the relationships between EHD-PRO scores with neurological disability, cognitive function, fatigue and health-related quality of life (HR-QOL).

**Results:**

Regardless of their phenotype, PwMS had significantly higher EHD-PRO scores than HCs. EHD-PRO scores did not differ between the two MS groups. EHD-PRO scores did not correlate with disability and fatigue scores, disease duration or cognitive z scores. In RRMS, the lack of emotional control was independently associated with a decrease in HR-QOL.

**Conclusion:**

The EHD-PRO is able to easily detect depressive mood and to differentiate between two clinical dimensions, emotional blunting and lack of emotional control. The scale is sensitive and seems robust to confounding factors. Lack of emotional control seems to contribute significantly to altered HR-QOL in RRMS.

## Introduction

Depressive mood (DM), anxiety and other emotional symptoms are more frequent in people living with multiple sclerosis (PwMS) than in the general population [1]. These symptoms can be related to factors unrelated to MS or to specific factors such as reactions to diagnosis, disability, or iatrogenic effects of treatments, as well as to immunological and neurological dysfunction [2]. Depression can have negative consequences on neurological care, adherence to medication or rehabilitation treatments [2]. Depression can also interfere with cognitive performance and ultimately contribute to a degraded health-related quality of life (HR-QOL) [3].

The identification and characterization of depressive symptoms, especially outside the context of major depressive disorder, may be difficult in neurological diseases in which confounding symptoms such as fatigue and cognitive symptoms can interfere with emotional assessment [2,4]. Some symptoms, such as apathy, anger, and emotional incontinence, can be attributed to depression, but they also belong to psycho-behavioural disorders associated with brain damage. Two scales have been reported to be less sensitive to somatic confounds, the Beck Depression Inventory-Fast Screen for Medical Patients (BDI-FS) [5] and the Hospital Anxiety and Depression Scale (HADS) [6]. These scales are useful for detecting major depression, but it is necessary to differentiate a symptom of depression from the syndrome of major depression and to decipher the sub-components of DM [4]. A DM scale, called “Echelle d’Humeur Depressive” (EHD) [7] has been developed. The focus of the EHD is not to determine a global score of depression; instead, it aims to characterize various emotional dimensions. It enables the identification of subjects who lack major depression or generalized anxiety disorder but who do have significant emotional disturbances. The original EHD consists of 18 items assessed by the examiner. The answer to each item is given using a 5-point Likert-type scale, from 0 (none) to 4 (severe). The authors performed principal components analysis, which revealed 5 factors: emotional blunting (EB), emotional incontinence, explosive mood/irritability, emotional lability and painful sadness [7]. From these factors the authors distinguished two clinical dimensions, emotional deficits and the lack of emotional control (EC) and proposed a dichotomy between depression in which inhibition predominates and depression in which anxious or agitated symptoms predominate [8]. Because patient-reported outcome (PRO) tools are easier to use in clinical practice and research, a self-report version of the EHD (EHD-PRO) has been developed (**EHD-PRO translated from French in S1 Text**) and validated in a sample of 77 MS patients, 97% of which with RRMS [9]. Patients were recruited in an outpatient MS clinic and the mean disease duration was 11.4 ± 8 years [9]. Principal components analysis confirmed a 2-factor structure that matched the two clinical dimensions of DM previously identified: EB, based on 7 items, which explained 33.5% of the variance; and EC, based on 4 items, which explained 20% of the variance. The questionnaire internal coherence coefficients (Cronbach alpha) were excellent for the whole scale (= 0.87) and the two sub-scales (0.89 for « blunted affect » dimension and 0.71 for « lack of emotional control » dimension) [9]. The questionnaire’s external validity was confirmed by a positive correlation between « lack of control » sub-score and state sub-score of the Stait-Trait Anger eXpression Inventory (r = 0.55, p<0.01) and correlation with the BDI score (r = 0.76, p<0.01; « lack of emotional control »/BDI: r = 0.68, p<0.01; « blunted affect »/BDI: r = 0.63, p<0.01) [9]. Test-retest reliability was good with a positive correlation between all the initial scores and their retests, a week later [9]. However, normative values in healthy subjects of the EHD-PRO were not established and correlations with disease parameters are unknown.

The aims of the present study were two-fold: First, to compare the EHD-PRO assessment and its two specific dimensions, lack of EC (EHD-EC) and EB (EHD-EB), between two samples of PwMS and a large sample of healthy controls (HC) to determine the prevalence of these emotional disorders in two clinical phenotypes of the disease, relapsing-remitting MS (RRMS) and primary progressive MS (PPMS); and second, to analyse the relationships between EHD-PRO scores with neurological disability, cognitive function, fatigue and HR-QOL.

## Materials and Methods

### Participants

The study population and eligibility criteria have been already described in detail [10]. All participants were required to be older than 18 years old and French speakers. Briefly, 60 PwMS with a diagnosis of RRMS [11] and 41 with a diagnosis of PPMS [12] were recruited between April 2009 and April 2011. HCs were divided into 20 groups according to five age categories, sex, and education level to be matched with PwMS. HCs received compensation for participating in the study.

The study was approved by the institutional review board of Bordeaux (CPP Bordeaux 2009/31). All subjects gave written informed consent prior to participation in the study.

### Assessment

Disability was measured using a French-adapted version of the Expanded Disability Status Scale (EDSS) [13].

#### Mood assessment

Each subject completed PRO questionnaires concerning mood (EHD-PRO), depressive symptoms (BDI-II [14] and BDI-FS [5], and anxiety, State-Trait Anxiety Inventory, State Form (STAI-S) [15] and received a fatigue score based on the UK Neurological Disability Scale [16].

The EHD-PRO scores include a total EHD-PRO score and two sub-scores (EHD-EC and EHD-EB). The EHD-EB score includes items 3, 6, 7 and 8, and the EHD-EC score includes items 1, 2, 4, 5, 9, 10 and 11. The EHD-PRO total score was the sum of the two dimensions of sub-scores.

According to the BDI-II, subjects were considered to be free of depressive symptoms if their BDI II score was less than 14 and to have moderate or severe depression if their score was above 20. The thresholds for the BDI-FS were 9 and above for moderate depression and 13 and above for severe depression. Abnormal fatigue or anxiety was defined by a score that was less than the 5th percentile of the HCs.

#### Health-Related Quality of Life (HR-QOL)

The French-validated version of the Short-Form 36 questionnaire [17] was used and two composite scores were calculated: the Physical Composite Score (PCS/SF-36) and the Mental Composite Score (MCS/SF-36) [17].

#### Neuropsychological assessment

PwMS and HCs were assessed with a comprehensive neuropsychological (NP) battery that has been described previously [10]. Briefly, it included the tests from the Brief Repeatable Battery–Neuropsychological [18], but the Computerized Speed Cognitive Test (CSCT) [19] was used instead of the Symbol Digit Modalities Test (SDMT) to assess information processing speed (IPS). The battery also included computerized sub-tests from the Test of Attentional Performance (TAP) [20] and some additional tests for executive functions, working memory and visuoconstruction [10]. Seven cognitive domains were categorized: IPS, attention, working memory, verbal and visual episodic memory, visuoconstruction, and executive function [10].

### Statistical analysis

Statistical analyses were performed with StatView version 5.0 software for Windows. Means and standard deviations (SDs) were calculated for age, disease duration, and for EHD-PRO and NP scores; medians and ranges were calculated for EDSS scores. Clinical characteristics and scores were compared using Chi-square, unpaired Student’s t or Mann-Whitney tests as appropriate.

Normative data were established from the mean and SD of the EHD-PRO scores of 415 HCs that were divided into different categories according to age and gender.

Z scores were calculated for each NP score and domain as previously reported.^10^ PwMS were considered cognitively impaired (CI) if their z scores were below the fifth percentile for their matched HC group and cognitively unimpaired (CU) if they did not.

The univariate correlations between each EHD-PRO and cognitive score and between EHD-PRO scores and age, gender, EDSS, UKNDS, BDI-FS, STAI-S and HR-QOL composite scores were calculated using Pearson’s test in each phenotype group. Factors not significant at the 0.05 level were removed by backward elimination. Two types of multivariate models were performed for each clinical phenotype. First, linear regression was performed to study the correlation of fatigue, anxiety and depression with EHD-PRO scores in each PwMS group. Age, gender and EDSS scores were used in the models. Second, linear regression was used to evaluate correlations between HR-QOL composite scores and EHD-PRO scores, taking other variables into account (age, gender, educational level, disease duration, EDSS score, fatigue and cognitive z scores). Only independent variables with a conservative significance level of p<0.25 in the univariate analysis were simultaneously entered in the multivariate models.

For all analyses, differences were considered significant when the p values were less than 5% except for univariate correlation analyses, where Bonferroni correction was applied.

## Results

### Characteristics of PwMS and HCs

Forty-one PwPPMS, 60 PwRRMS, and 415 HCs were included in this study. Table 1 shows the clinical characteristics of the PwMS. Based on their demographic and clinical characteristics, the PwPPMS and PwRRMS were compared with 263 and 310 HCs, respectively. Thirty-six PwPPMS (87.8%) and 53 PwRRMS (88.3%) were taking disease-modifying drugs at the time of the examination. Among all the clinical characteristics, EDSS score, age, and gender differed significantly between the two groups of PwMS (p<0.001, p<0.001 and p<0.05, respectively).

**Table 1:** Clinical and PRO scores of PwMS.

	RRMS group	PPMS group
n	60	41
Gender (F/M)	49/11^ns^	24/17^ns^
Age (years)	37.3±9.9^ns^	52.1±8.7^ns^
Educational level (≥baccalaureate/<baccalaureate)	38/22 ^ns^	19/22 ^ns^
Disease duration (years)	4.1±3.0	4.8±3.9
EDSS scores	1.5 (0–4.5)	3.5 (1.5–7.0)
BDI scores	11±7.9***	13.5 ±7.2***
BDI-FS scores	3.3±1 ***	3.7±2.8***
STAI-S	35.5±10.8***	34.3±9.2**
Fatigue scores	1.1±1.4***	1.4±1.3***
EHD-PRO total score	19.4±6***	19.2±6.3***
EHD-EC lack of emotional control	13.5±4.6***	12.9±4.7***
EHD-EB emotional blunting	5.9±2.1**	6.3±2.2***
Proportion of PwMS with moderate to severe depression (BDI)	15	24.4
Proportion of PwMS with moderate to severe depression (BDI-FS)	10	7.3
Proportion of PwMS with anxiety (STAI-S)	33.3	19.5
Proportion of PwMS with emotional blunting (EHD EB)	23.3	31.7
Proportion of PwMS with decrease emotional control (EHD-EC)	23.3	26.8
Proportion of PwMS with decreased emotional control or with emotional blunting	33.3	41.5
PCS/SF-36	64.4±18.4***	48.5±12***
MCS/SF-36	64.5±18.5***	57.7±15.3***

F: female; M: male. RRMS, relapsing–remitting multiple sclerosis; PPMS, primary progressive MS. For all clinical data, scores are expressed as the mean ± standard deviation (SD), except for EDSS, which are expressed as the median (range).

EDSS: Expanded Disability Status Scale; BDI: Beck Depression Inventory II; BDI-FS: Beck Depression Inventory-Fast screen; STAI-S: State Anxiety scale of State-Trait Anxiety Inventory-State Form Y; EHD-PRO: Echelle d’Humeur Dépressive-Patient-reported outcomes; EHD-EC: Echelle d’Humeur Dépressive lack of Emotional Control-Patient Reported Outcomes; EHD-EB: Echelle d’Humeur Dépressive Emotional Blunting-Patient Reported Outcomes; EHD-PRO: total score corresponding to an addition of EHD-EC and EHD-EB; PCS/SF-36 MCS/SF-36. p values from a chi-squared test comparing sex; p values from non-matched t-tests comparing means; p values between each MS group and matched controls, 60 PwRRMS and 310 matched controls, 41 PwPPMS and 263 matched controls: ^ns^,non significant

*<0.05

**<0.01

***<0.001.

#### Comparison of EHD-PRO scores and other PRO scores between PwMS and HCs

The EHD-PRO scores and other PRO results for the two PwMS groups and their respective matched HCs are presented in Table 1. Significant differences in clinical and PRO scores between each MS phenotype and the matched HCs are presented in Table 1. Regardless of group, PwMS had significantly higher scores on the EHD-PRO and its two dimensions as well as on all PRO (depression, anxiety, fatigue and HR-QOL) compared to their matched HCs.

#### EHD-PRO scores and other PRO scores, according to MS phenotype

EHD-PRO scores and other PRO scores did not differ between the RRMS group and the PPMS group, except for the physical composite score of the SF-36. No significant difference was found between the proportion of PwRRMS and PwPPMS with moderate-to-severe depression detected by the BDI-II or the BDI-FS. Similarly, no significant difference was found between the proportion of PwRRMS and PwPPMS identified as having moderate-to-severe depression when these two scales were applied separately or together. The proportion of anxious patients did not differ between the two groups.

#### Detection of depressive mood disorders with the EHD-PRO in PwMS with moderate and severe depression or without depression

Fifteen percent of PwRRMS and 24.4% of PwPPMS were diagnosed with moderate or severe depression by the BDII but 33.3% of PwRRMS and 415% of PwPPMS had at least one abnormal EHD-PRO score. Among the PwMS with moderate or severe depression according to the BDI-II, 88.9% (RRMS) and 90% (PPMS) of them had at least one abnormal EHD-PRO score. All PwMS in the group with moderate-to-severe depression according to the BDI-FS had abnormal EHD-PRO scores. However, 14.6% of the PwPPMS had at least one abnormal EHD-PRO score, but normal BDI-II, BDI-FS or STAI scores.

### Normative data of EHD-PRO scores are presented in S1 Table. EHD-PRO and other PRO scores, according to cognitive impairment

The proportion of PwMS who were impaired in at least one cognitive domain (CI) was 25.4% in the RRMS group and 47.3% in the PPMS group (p<0.05). total EHD-PRO score and sub-scores did not differ significantly between CI and CU PwMS. BDI-II, BDI-FS, anxiety and fatigue scores also did not differ between CI and CU PwMS. There was no significant correlation between any of the EHD-PRO scores and z scores of each cognitive domain in the RRMS and PPMS groups.

### Association of EHD-scores with demographic and clinical variables

Among the characteristics of age, sex, and educational level, only gender was correlated (p<0.05) with the EHD-PRO scores, but this correlation was no longer significant after applying Bonferroni correction.

EHD-PRO scores did not correlate with EDSS and fatigue scores or with disease duration in the RRMS or PPMS group. BDI-II scores did not correlate with fatigue.

#### Multivariate regression analysis

The results of multivariate regression analyses performed to verify the association of EHD-PRO scores with the demographic and clinical variables are presented in Tables 2 and 3. For each EHD-PRO score, the variables entered in the multivariate model were age, gender, and EDSS, BDI-FS, STAI-S, and fatigue scores.

**Table 2:** Linear regression model describing EHD scores in PwRRMS.

EHD scores	independent variables included in model ^b^	R univariate analyses	p values univariate analyses	Adjusted R^2^ model	p values multivariate analyses
**EHD-PRO total score**	Age	0.117	0.38	**0.498**	**<0.0001**
	**Gender** ^a^	**0.306**	**<0.05**		
	EDSS	0.107	0.42		
	Fatigue	0.013	0.92		
	**BDI-FS** ^a^	**0.550**	**<0.001**		
	**STAI-S** ^a^	**0.599**	**<0.001**		
**EHD-HC lack of emotional control**	Age	0.049	0.71	**0.461**	**<0.0001**
	**Gender** ^a^	**0.278**	**<0.05**		
	EDSS	0.083	0.53		
	Fatigue	0.015	0.90		
	BDI-FS	0.494	<0.001		
	**STAI-S** ^a^	**0.636**	**<0.001**		
**EHD-EB emotional blunting**	Age	0.230	0.78	**0**.**249**	**<0.0001**
	Gender	0.270	<0.05		
	EDSS	0.126	0.34		
	Fatigue	0.004	0.98		
	**BDI-FS** ^a^	**0.499**	**<0.001**		
	STAI-S	0.328	<0.01		

^a^ variables significantly correlated with EHD scores in multivariate analyses.

^b^ variables with a p value <0.25 in univariate analyses. Age, gender, EDSS score were included in multivariate analyses.

RRMS: relapsing–remitting multiple sclerosis; EDSS: Expanded Disability Status Scale; BDI-FS: Beck Depression Inventory-Fast Screen; S-Anxiety: State Anxiety scale of State-Trait Anxiety Inventory-State Form Y; EHD-EC: Echelle d’Humeur Dépressive lack of Emotional Control-Patient Reported Outcomes; EHD-EB: Echelle d’Humeur Dépressive Emotional Blunting-Patient Reported Outcomes; EHD-PRO: total score corresponding to an addition of EHD-EC and EHD-EB.

**Table 3:** Linear regression models describing EHD scores in PwPPMS.

EHD scores	independent variables included in model	R univariate analyses	p values univariate analyses	Adjusted R^2^ model	p values multivariate analyses
**EHD-PRO total score**	Age	-0.224	0.16	0.677	**<0.0001**
	Gender	0.033	0.84		
	EDSS	-0.322	<0.05		
	Fatigue	-0.270	0.87		
	**BDI-FS** ^a^ ^**,**^ ^b^	**0.735**	**<0.001**		
	**STAI-S** ^a^ ^**,**^ ^b^	**0.707**	**<0.001**		
**EHD-EC lack of emotional control**	Age	-0.244	0.13	**0.668**	**<0.0001**
	Gender	-0.24	0.88		
	EDSS	-0.275	0.08		
	Fatigue	0.009	0.96		
	**BDI-FS** ^a^ ^**,**^ ^b^	**0.72**	**<0.001**		
	**STAI-S** ^a^ ^**,**^ ^b^	**0.715**	**<0.001**		
**EHD-EB emotional blunting**	Age	-0.130	0.42	**0.494**	**<0.0001**
	Gender	0.145	0.37		
	EDSS	-0.345	<0.05		
	Fatigue	0.048	0.77		
	**BDI-FS** ^a^ ^**,**^ ^b^	**0.600**	**<0.001**		
	STAI-S ^a^	0.527	<0.001		

^a^ variables significantly correlated with EHD scores in multivariate analyses.

^b^ variables with a p value <0.25 in univariate analyses. Age, gender, EDSS score were included in multivariate analyses.

PPMS: primary progressive multiple sclerosis. EDSS: Expanded Disability Status Scale; BDI-FS: Beck Depression Inventory-Fast Screen; S-Anxiety: State Anxiety scale of State-Trait Anxiety Inventory-State Form Y; EHD-EC-PRO: Echelle d’Humeur Dépressive lack of Emotional Control-Patient Reported Outcomes; EHD-EB-PRO: Echelle d’Humeur Dépressive Emotional Blunting-Patient Reported Outcomes; EHD-PRO: total score corresponding to an addition of EHD-EC and EHD-EB.

In the RRMS, the EHD-PRO score was predicted independently by gender and by BDI-FS and STAI-S scores (p<0.001; R^2^ = 0.498). The EHD-EC score was predicted independently by gender and anxiety score but not by BDI-FS score (p<0.001; R^2^ = 0.461), whereas the EHD-EB score was predicted independently only by the BDI-FS score (p<0.001; R^2^ = 0.249).

In the PPMS, the multivariate linear regressions showed significant correlations between each EHD score and the BDI-FS score. BDI-FS and STAI-S scores were significantly associated with EHD-PRO total score (p<0.001; R^2^ = 0.677) and with EHD-EC score (p<0.001; R^2^ = 0.668), whereas the EHD-EB score was only significantly correlated with the BDI-FS score (p<0.001; R^2^ = 0.494).

### HR-QOL

Tables 4 and 5 present the results of the multivariate regression analyses performed to identify the determinants of HR-QOL, including demographic and clinical variables and the EHD-PRO scores. In the PwRRMS, the EHD-EC score was retained in the final models to explain each HR-QOL sub-score. EDSS and fatigue scores also explained PCS/SF-36 score, and fatigue also explained MCS/SF-36 score. In the PwPPMS, no variables were maintained in the final model.

**Table 4:** Multivariate analyses of HR-QOL in RRMS patients (models including EHD PRO-subscales).

HR-QOL scores	independent variables included in model	R univariate analyses	P values Univariate analyses	Adjusted R^2^ model	P values multivariate analyses
**PCS/SF-36**	Age	0,134	0.31	**0.537**	**<0.001**
	Gender ^a^	0.216	0.10		
	EL^a^	0.248	0.06		
	**EDSS** ^b^	**-0.320**	**0.01**		
	**Fatigue** ^a^ ^**,**^ ^b^	**-0.555**	**<0.001**		
	**EHD-EC** ^a^ ^**,**^ ^b^	**-.326**	**0.01**		
	EHD-EB^a^	-0.256	0.05		
	z score executive functions^a^	-0.176	0.19		
	z score visuo-construction^a^	0.168	0.21		
**MCS/SF-36**	Age	-0.081	0.54	**0.511**	**<0.001**
	Gender ^a^	-0.174	0.19		
	EDSS	-0.057	0.67		
	**Fatigue** ^a^ ^**,**^ ^b^	**-0.211**	**0.11**		
	**EHD-EC** ^a^ ^**,**^ ^b^	**-0.688**	**<0.001**		
	EHD-EB^a^	-0.384	<0.01		
	z score visuo-construction^a^	-0.157	0.24		

^a^ variables significantly correlated with EHD scores in multivariate analyses.

^b^ variables with a p value <0.25 in univariate analyses. Age, gender, EDSS score were included in multivariate analyses.

RRMS, relapsing–remitting multiple sclerosis. HR-QOL: Health-Related Quality of life; PCS/SF-36: Physical Composite Score of the Short-Form-36 MCS/SF-36: Mental Composite Score of the Short-Form-36; EHD-EC: Echelle d’Humeur Dépressive lack of Emotional Control-Patient Reported Outcomes; EHD-EB: Echelle d’Humeur Dépressive Emotional Blunting-Patient Reported Outcomes.

**Table 5:** Multivariate analyses of HR-QOL in PPMS patients (models including EHD PRO-subscales).

HR-QOL scores	independent variables included in model	R univariate analyses	P values Univariate analyses	Adjusted R^2^ model	P values multivariate analyses
PCS/SF-36	Age	-0.096	0.56	0.074	(0.09) ^ns^
	Gender	-0.066	0.69		
	EDSS^a^	-0.272	0.09		
	EHD-EC	0.151	0.36		
	EHD-EB	0.102	0.54		
	z score visual memory ^a^	-0.204	0.21		
	z score visuo-const ^a^	-0.204	0.21		
MCS/SF-36	Age ^a^	0.260	0.10	0.223	0.008
	Gender	-0.156	0.33		
	EDSS ^a^	0.254	0.11		
	EHD-EC ^a^	-0.409	<0.01		
	EHD-EB ^a^	-0.450	<0.01		
	z score IPS ^a^	0.259	0.12		
	z score attention ^a^	0.195	0.24		
	z score verbal memory ^a^	0.212	0.19		

^a^ variables significantly correlated with EHD scores in multivariate analyses.

^b^ variables with a p value <0.25 in univariate analyses. Age, gender, EDSS score were included in multivariate analyses

^ns:^ non significant

PPMS, primary progressive multiple sclerosis. HR-QOL: Health-Related Quality of life; PCS/SF-36: Physical Composite Score of the Short-Form-36 MCS/SF-36: Mental Composite Score of the Short-Form-36; EHD-EC: Echelle d’Humeur Dépressive lack of Emotional Control-Patient Reported Outcomes; EHD-EB: Echelle d’Humeur Dépressive Emotional Blunting-Patient Reported Outcomes.

When performing the same multivariate analyses with the same variables but replacing the two sub-scores with the EHD-PRO total score, the EHD-PRO total score, fatigue score and EDSS score correlated significantly with PCS/SF-36 score. EHD-PRO total score was the only variable significantly correlated with MCS/SF-36 score in the RRMS group. In the PPMS group, only EHD-PRO total score remained in the final model with MCS/SF-36 score (data not shown).

## Discussion

Identifying depression and emotional disturbances in PwMS is a clinical challenge. These symptoms are frequent in MS and have important consequences for the daily life of PwMS. In the present study, depression scores (using the BDI), BDI-FS scores, anxiety scores and EHD-PRO total scores and sub-scores differed significantly between the HCs and the two samples of PwMS. The EHD-PRO has been developed to evaluate the main dimensions of depressed mood, emotional deficits and the lack of emotional control, which can be observed in MS [9]. This study, confirms that this self-report tool is easy to use in PwMS and is able to easily distinguish these two dimensions. We observed high proportions of PwRRMS (33.3%) and PwPPMS (41.5%) with an abnormal score on at least one of the two sub-scores. The proportion of PwMS identified as having moderate or severe depression by the BDI or the BDI-FS did not exceed 24.4%. In the subgroup of PwRRMS and PwPPMS with moderate or severe depression according to the BDI-II, the EHD-PRO detected 88.9% of the PwRRMS and 90% of the PwPPMS with depressive mood disorders. All of the PwPPMS detected by the BDI-FS were also detected by the EHD-PRO. These results highlight the sensitivity of this scale to moderate-to-severe depressive disorders in MS. Moreover, 14.6% of the PwPPMS had at least one abnormal EHD-PRO score, but they were not diagnosed by the BDI-II or BDI-FS or by the STAI. This demonstrates that the EHD-PRO may be able to detect not only moderate to severe depression but also subtle elements of DM and this can be important of the clinical management of the patients. Similar results have been shown in a sample of patients with Alzheimer’s disease, in which a significant proportion of the patients met the criteria for dysthymic disorder but not for major depression or generalized anxiety disorder [21].

To our knowledge, this study is the first to compare emotional dimensions such as EB or lack of EC in two MS phenotypes. No EHD-PRO scores differed significantly according to MS phenotypes, suggesting that the EHD-PRO could detect DM independently of the phenotype at the onset of the disease. Similar results were observed for each other PRO scores, except for the physical composite score of the SF-36; this score was higher in the PwRRMS, although this can be attributed to a difference in disability.

One limitation of emotional assessments in MS is the interference of neurological confounds. Interestingly, the EHD-PRO scores did not correlate with either the EDSS and fatigue scores or with disease duration in both groups. No demographic variables influenced the EHD-PRO scores. Moreover, we did not observe correlations between EHD-PRO scores and cognitive z scores in the two samples of PwMS. This suggests that the EHD-PRO is not very influenced by somatic or demographic factors. Interestingly lack of EC and cognitive z scores did not correlate. This suggests that EHD-PRO may help to identify the lack of EC related to DM which is different to the lack of EC, related to emotional processing impairment. Indeed, several studies evaluated social cognition in MS [22], and in particular emotional processing which refers to perceiving and using emotions. The EHD allows the identification and the characterization of emotional disturbances in relation with DM. It could be very important in the management of cognitive and emotional problems of MS patients. If the lack of EC is in relation with mood symptoms a psychological intervention could be proposed. If it is due to cognitive problems, cognitive management has to be considered.

In the RRMS group, the results suggest a dichotomy between the two dimensions of DM assessed by the EHD-PRO: the lack of EC, which is mainly associated with anxiety; and EB, which is mainly associated with depression. The EHD-PRO total score reflects both depression and anxiety. We found similar associations in the PPMS group: anxiety was associated with the lack of EC, corresponding to anxious agitation, anger, impulsivity or hyperemotivity [7–9], but was not associated with EB, which refers to deficits in the ability to feel and express emotions and includes affective blunting, anhedonia, loss of initiative or motivation, prosodic flattening and psychomotor slowing. The EHD-PRO total score was predicted by both depression and anxiety. Fatigue did not remain in the final models. The BDI-II scores did not correlate also with the fatigue score. This is possibly due to a lack of sensitivity of the fatigue score used in this study to the mental dimension of fatigue. Depression assessed by the BDI-FS was associated with each EHD score. In the RRMS group, gender was associated with the EC and total EHD scores. It has been previously shown that EC scores are higher in women than in men in MS [9], possibly because women express more of their emotions.

The validity of the EHD-PRO is reinforced by the correlations with the HR-QOL composite scores. The EHD-EC score was the main determinant of the mental HR-QOL composite score in the RRMS patients. Indeed, lack of EC was the dimension retained with fatigue and EDSS scores to explain the physical dimension of HR-QOL and was retained with fatigue score to explain the mental dimension of HR-QOL in PwRRMS. Fatigue and disability are well-known factors that have been implicated in HR-QOL [23]. The lack of EC seems to be a prominent factor in HR-QOL in PwMS. This dimension needs further study to determine the nature of the underlying processes involved and to identify possible interactions with other impaired functions in the disease.

This study has some limitations. The absence of MRI analysis precludes interpretation of underlying mechanisms. From a methodological point of view, an assessment using the trait form of the STAI would be pertinent to explore the predictions for HR-QOL.

## Conclusion

The use of the EHD-PRO to assess emotional states in MS is promising. This self-administered questionnaire can be easily provided by caregivers. Our findings suggest that the EHD-PRO can evaluate DM independently of clinical symptoms such as cognitive disorders, fatigue or physical disability. The EHD-PRO can detect patients with DM, even some who have only subtle mood change, but also can refine, the diagnosis of DM and can identify two dimensions, EB and lack of EC. Lack of EC may reflect emotional instability that is more closely related to neurological disease independent of any depression or that is related to DM. This study demonstrates that the lack of EC has a significant impact on HR-QOL in PwMS. Therefore the diagnosis of lack of EC due to DM could very useful for the clinical management of PwMS. The EHD-PRO may also be useful for the monitoring of pharmacological, behavioural and/or rehabilitation therapies.

## Supporting Information

S1 Table Normative data of EHD-PRO scores in Healthy Controls. (DOC)

S1 Text Echelle d’Humeur Dépressive-Patient Related Outcome (EHD-PRO, translated from French). (DOC)
