# Supplementary material for: Deciphering Depressive Mood in Relapsing-Remitting and Progressive Multiple Sclerosis and Its Consequence on Quality of Life
Source: PLoS One. 2015 Nov 10;10(11):e0142152. doi: 10.1371/journal.pone.0142152 (PMC4640551; doi:10.1371/journal.pone.0142152)
Supplement: S1 Table — (DOC) [file pone.0142152.s001.doc]

S1 Table: Normative data of EHD-PRO scores in Healthy Controls

|  | | | EHD-PRO scores | | | | | |
| --- | --- | --- | --- | --- | --- | --- | --- | --- |
|  |  |  | EHD-PRO  total score | | EHD-EC | | EHD-EB | |
| Age category | Sex | Size (n) | Mean | SD | Mean | SD | Mean | SD |
| 18-34 | H | 50 | 16 | 3.9 | 10.2 | 3.2 | 5.9 | 1.7 |
|  | F | 50 | 17 | 4.6 | 11.8 | 4.2 | 5.2 | 1.5 |
| 35-54 | H | 86 | 15.3 | 3 | 10.2 | 2.6 | 5.1 | 1.2 |
|  | F | 92 | 13.3 | 3.5 | 11.3 | 3 | 5.1 | 1.2 |
| > 55 | H | 46 | 15.1 | 3.2 | 9.8 | 2.7 | 5.3 | 1.2 |
|  | F | 91 | 16.5 | 4.5 | 11.2 | 3.6 | 5.3 | 1.6 |

F: female; M: male. All scores are expressed as mean ± standard deviation (SD). Normative based on 415 HCs were established according to three age categories (18–34, 35-54 and ≥ 55 years) and gender. There was no significant difference according to the level of education. EHD-EC: Echelle d’Humeur Dépressive lack of Emotional Control-Patient Reported Outcomes; EHD-EB: Echelle d’Humeur Dépressive Emotional Blunting-Patient Reported Outcomes; EHD-PRO: total score corresponding to an addition of EHD-EC and EHD-EB
