# Supplementary material for: Deciphering Depressive Mood in Relapsing-Remitting and Progressive Multiple Sclerosis and Its Consequence on Quality of Life
Source: PLoS One. 2015 Nov 10;10(11):e0142152. doi: 10.1371/journal.pone.0142152 (PMC4640551; doi:10.1371/journal.pone.0142152)
Supplement: S1 Text — (DOC) [file pone.0142152.s002.doc]

S1 Text : Echelle d’Humeur Dépressive-Patient Related Outcome (EHD-PRO, translated from French):

Answer the following questions taking into account your state over the last week.

Four possible responses: not at all, moderately, quite a lot, very Much.

1. Do you feel that you let your emotions show too much (tears, shouts, looking scared, etc)?

2. Do you feel that you rapidly change mood and for little or no reason (for instance changing from happiness to sadness or quietness to angriness)?

3. Do you feel that you have difficulties to show your feelings to others (both with words and gestures)?

4. Do you feel that you have difficulties to control your emotions (for instance, to be overcome by sadness or despair or angriness)?

5. Do you feel sad, unhappy?

6. Do you feel that you are unconcerned, that you have difficulties to feel anything whether pleasure or displeasure?

7. Have you got difficulties to enjoy sounds, odours, tastes, colours you were sensitive to before?

8. Do you feel that you are in a monotonous mood, always the same?

9. Do you feel that you are more emotive than usual, that you have intense emotional responses (tears, anger, panic, etc)?

10. Do you feel irritable, nervous?

11. Do you feel that you lose your temper easily, that you have fits of anger?

EHD-PRO total score corresponds to an addition of EHD-EC and EHD-EB scores:

- lack of emotional control items (EHD-EC): 1,2,4,5,9,10,11

- emotional blunting items (EHD-EB): 3,6,7,8
